# Case of Phthisis Pulmonalis, Cured by Mercury

**Published:** 1808-03

**Authors:** William Harris


*Case of Phthisis Pulmonalis, cured by Mercury; by* Dr. William Harris. *In a Letter to* Dr. Rush.

Dear Sir,

It is with heart-felt joy, I now have the satisfaction of informing you of the salutary effect of your prescription in pulmonary consumption, the subject of which is myself. Last spring, I was attacked with phthisis pulmonalis, from being exposed thoughtlessly to a current of air, when in a full perspiration from exercise in the garden; I was in a great heat, and the sweat dripping from me when I opened my breast, bare to the fresh breeze then blowing; the effect was a sudden chill, pain in my breast, wheezing, cough, and soon after expectoration, with copious night sweats, which wore me down to a mere skeleton. I had no prospect of subduing the disease, but looked forward for it to end only with my life; when, about three weeks since, I was affected, after being exposed to cold, with a violent peripneumony, from which I had little prospect of recovery. In this situation your letters occurred to me, written in the Medical Repository. I was determined at all events to try the effect: danger from the violence of the disease would not admit the gradual process of the calomel; I took it as copiously as I could bear it, and rubbed on the mercurial ointment on my side and thighs, at the same time bled copiously, 30 ounces a-day, for several days; blistered the whole breast, depleted in every possible manner, to give the mercury liberty to operate; in 48 hours it answered the purpose; as soon as my mouth got got sore, the pains gradually left me, cough ceased, expectoration abated, and every pulmonary symptom began to disappear. I had expectorated nearly, in some days, half a pint, and coughed incessantly; now, I cough none of any consequence, and do not expectorate more than two or three times in the day, and that with ease; my appetite has returned, and every prospect of health is great.

Whether I should continue the ptyalism, or use tonics, I am rather at a loss to know; my debility is very great, not being able yet to walk alone. I can sit up a little. From the violence of the peripneumony in a great measure, I carried the spitting to upwards of two quarts daily for some time; now I do not spit more than half a pint, and feel quite well; and should I not get cold, I believe no doubt remains, but that the mercury has conquered the disease, which I believe nothing else yet known could have done; and for the discovery and publication of which, I acknowledge myself indebted to you for the rescue of my life.

*Belle-Fort, September* 20, 1803.

